# Current Advances in the Use of Tissue Engineering for Cancer Metastasis Therapeutics

**DOI:** 10.3390/polym16050617

**Published:** 2024-02-23

**Authors:** Preeya D. Katti, Haneesh Jasuja

**Affiliations:** 1 American University of Caribbean, Miramar, FL 33025, USA; 2 Sanofi, Boston, MA 02142, USA; haneeshjasuja@gmail.com

**Keywords:** tissue engineering, cancer, metastasis, in vitro models, 3D models

## Abstract

Cancer is a leading cause of death worldwide and results in nearly 10 million deaths each year. The global economic burden of cancer from 2020 to 2050 is estimated to be USD 25.2 trillion. The spread of cancer to distant organs through metastasis is the leading cause of death due to cancer. However, as of today, there is no cure for metastasis. Tissue engineering is a promising field for regenerative medicine that is likely to be able to provide rehabilitation procedures to patients who have undergone surgeries, such as mastectomy and other reconstructive procedures. Another important use of tissue engineering has emerged recently that involves the development of realistic and robust in vitro models of cancer metastasis, to aid in drug discovery and new metastasis therapeutics, as well as evaluate cancer biology at metastasis. This review covers the current studies in developing tissue-engineered metastasis structures. This article reports recent developments in in vitro models for breast, prostate, colon, and pancreatic cancer. The review also identifies challenges and opportunities in the use of tissue engineering toward new, clinically relevant therapies that aim to reduce the cancer burden.

## 1. Introduction

Cancer is emerging as a leading cause of premature death worldwide [1]. Globally, the World Health Organization reported 9.6 million deaths in 2018, making cancer the second leading cause of death worldwide. The large global economic burden of cancer from 2020 to 2050 was estimated to be USD 25.2 trillion [2]. Metastasis remains the primary cause of death due to cancer [3]. In a 2022 study, it was estimated that 623,405 people were living with metastatic cancers or metastatic melanoma in the US, and that number is expected to increase to 693,452 by the year 2025 [4]. A 2007 study reported that the economic burden for patients with metastatic bone diseases is USD 12.6 billion. This number represents 17% of the total medical burden estimated by the National Institutes of Health [5]. A more recent 2022 study estimated that the bone metastasis burden has increased to 20% of the overall oncology costs [6]. Alarmingly, most survivors with metastatic cancer of various types, except melanoma, have a life expectance of less than five years [4]. Palliative treatments are often the only course of treatment for metastasis. For example, at bone metastasis, palliative treatment includes the use of drugs, such as bisphosphonates and Denosumab, which are used to stabilize skeletal issues [7,8]. The complex cascade of biochemical and resulting pathological events that lead to metastasis to bone is still mostly unknown. This knowledge is further hampered by the lack of appropriate and relevant model systems for testing new drugs and therapies. In vitro models mimicking bone metastasis are much needed for this purpose.

**Figure 1:**
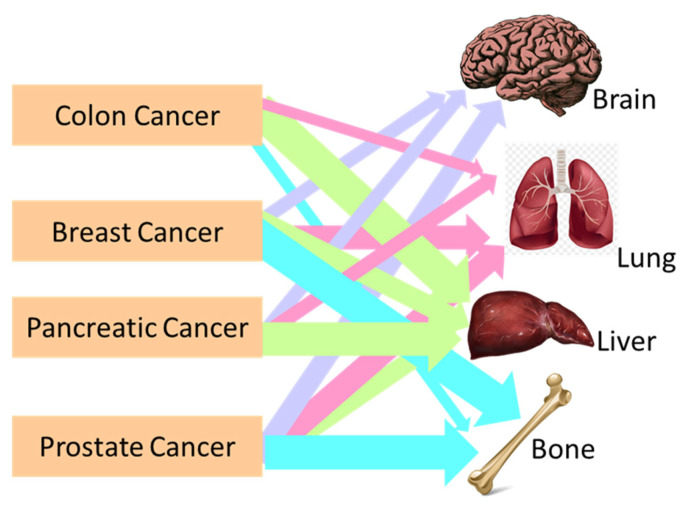
Schematic representation showing the metastasis locations of colon, breast, pancreatic, and prostate cancer in the human body.

All types of cancers can spread. The mechanisms of spreading cancer are varied. These range from cancer invading normal tissue in its proximity, traveling through either the lymphatic system or blood vessels to other parts of the body, attaching to invading blood vessel walls and forming tumors, to the growth of new blood vessels that enable metastatic tumor growth. Different cancers have the propensity to metastasize to specific locations in the body, e.g., breast cancer and prostate cancer tend to spread to the bones, and colon cancer and pancreatic cancer spread to the liver, as shown in Figure 1. Metastasis is the cause of most patient deaths, yet the mechanisms of metastasis remain primarily unknown. The process is fundamentally described as a two-step process, with the initial dislocation of cancer from its primary site and transportation of cells to a distant site through blood and lymph systems, and the phenomenon of recolonization of cancer at the remote site [9].

Tumorigenesis has been traditionally studied in 2D cultures of cancer cells. Although many characteristics of cancer cells have been well explored using these 2D cell culture systems, they do not replicate the realistic tumor microenvironment. The 3D culture systems have been increasingly gaining attention for the past few years. These systems include 3D spheroids of cancer cells. Extensive studies report using 3D spheroid models of cancer [10], and their important use in screening drugs. The spheroids are fundamentally clusters of cancer cells that are grown either in suspension or embedded in a matrix. Although the spheroids have 3D structures, they do not accurately capture the mechanical, biological, and chemical characteristics of the metastasis site. While extensive studies are undertaken on the development of the complex chemo-physio-mechanical analogs of the primary site of cancer [11], efforts in producing accurate 3D models of metastasis are rare, as they do not represent the migration characteristics of metastasis well.

Further, transwell-based assays are useful to assess the inherent migratory and invasion characteristics of cancer cells [12,13,14]. Migration is an important characteristic of cancer cells that are likely to arrive at the metastatic site—they do not capture the behavior of the cells at metastasis. Several detailed reviews illustrate the important use of transwell assay-based models [15]. To develop a deeper understanding of the process of cancer metastasis and to be able to aid in the development of effective therapies for metastasis prevention and cure, in vitro and in vivo models are extensively investigated. While in vivo models can capture the complexity of living systems and are generally considered useful models for studying primary cancer types, they often fail to develop into cancer metastasis.

Robust in vitro models present themselves as a fast, inexpensive evaluation route for the study of cancer metastasis. The extremely high-cost and time-intensive nature of PDX models further necessitates robust in vitro systems that effectively represent cancer and metastasis stages. Many in vitro models have been developed for capturing various aspects of tumor growth, proliferation, invasion into tissues, intra and extravasation through blood vessels, angiogenic characteristics, and also delivery of drugs and their efficacies [15]. The source of cancer cells, either commercial or patient-derived origins, for seeding the in vitro models is just as important. Many commercial cell lines of several cancer types are easily available and are used to study tumor cell biology and proliferation [16,17]. The molecular profiles and various characterizations of human cancer cell lines are available in the Cancer Line Encyclopedia [10,18,19,20]. Often, the spectrum of variabilities within a particular cancer type is very vast, and the commercial cell lines are not able to capture such a wide spectrum of variabilities and, thus, achieve limited clinical relevance for patients [21]. Patient-derived cell lines are thus increasingly popular as a source in the development of in vitro cancer models. While cell lines from patients, even with advanced cancer stages, are available [22], the availability of cancer cell lines from metastasized tissue types is rare. This review captures the development of metastasis tumor models of various cancer types and can help assist in the development of appropriate metastasis models for specific cancer types.

## 2. Tissue Engineering

**Figure 2:**
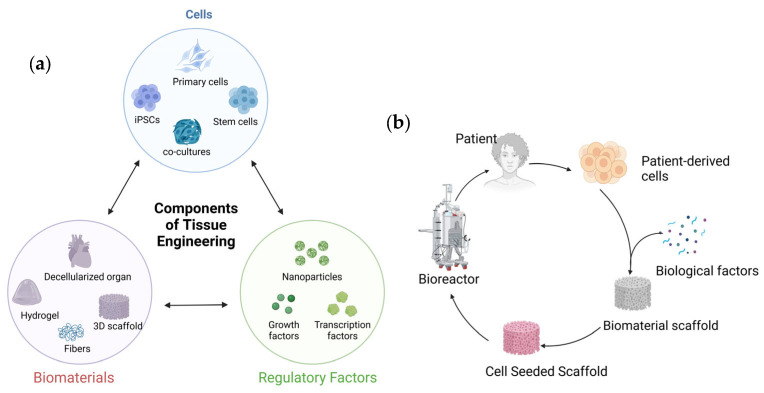
Schematic showing tissue engineering: (**a**) three important components of tissue engineering and (**b**) the tissue engineering process.

Tissue engineering provides an important platform for the design of site-specific structural and biological similarity of metastasis. Tissue engineering is an interdisciplinary field that aims to develop new tissue and organ substitutes using biological sciences and engineering. The primary components of tissue engineering include biomaterials and scaffolds, cells, and regulatory signals, such as the use of growth factors (Figure 2).

Originally proposed by Langer and Vacanti [23], it remains the forefront technology for regenerative medicine. The three important components of tissue engineering are the cells, regulatory signals, such as growth factors, and biomaterials, such as scaffolds (Figure 2a). The scaffolds are designed with porous microstructures and made of degradable materials. When seeded with appropriate human cells in the presence of growth factors, it enables the formation of engineered tissues, while the scaffolds themselves degrade. The development of in vitro models of cancer metastasis benefits from advances in tissue engineering of brain, lung, liver, and bone tissue. Tissue engineering has the potential to transform cancer research by providing mechanisms to observe tumorigenesis and migration at metastasis directly. Inducing angiogenesis is an important hallmark of cancer [24,25] and, thus, growth of solid tumors is often associated with neovascularization. Tissue engineering also provides the ability to recapitulate the tumor microenvironment and its complex and multifold characteristics [26]. The phenomenon of neovascularization around tumors was observed over a 100 years ago [27]. Thus, the importance of angiogenesis for tumor development also makes the tissue-engineered scaffolds with engineered porosities a promising platform for studying cancer metastasis. Thus, tissue-engineered constructs can also be used to study angiogenic characteristics of tumor growth factors. Hypoxic conditions of the tumors lead to the production of pro-angiogenic factors that enable neovascularization in the tumors. VEGF is an important angiogenic factor that plays a vital role in tumor growth as well as metastasis [28]. Hence, many antiangiogenic drugs (AAD) are used clinically. The exact mechanics of the AAD in controlling metastasis remain unknown [29]. Hence, VEGF-loaded scaffolds are extensively investigated for bone regeneration [30,31]. Scaffolds are used to evaluate the role of angiogenic factors in tumorigenic properties [32]. Cancer cells also secrete other growth factors and cytokines, such as basic fibroblast growth factor and interleukin-8, which also promote blood vessel formation and can thus be incorporated into the scaffolds.

The tissue engineering approaches toward understanding cancer have been studied extensively over the last two decades [33], enabling the advent of bioengineered tumors that represent viable in vitro models of cancer [34,35,36] using many biomaterial constructs [37,38,39,40]. It is expected that the next-generation 3D tissue-engineered constructs may replace animals in cancer drug testing [41]. The specific role of the metastasis site microenvironment is an important area of scientific investigation [42]. Recent advances in regenerative medicine pave the way for new cancer therapeutics as well as methodologies for the evaluation of fundamental cancer biology at metastasis [43,44].

In addition, in recent years, the development of bioreactors that provide important mechanical cues through fluid-enabled shear stresses has brought further development in viable in vitro models [45,46,47,48]. The metastasis condition is often delayed, as clinically, cancer cells are reported to remain dormant at the distant site after the removal of the tumor from the primary site. Tissue-engineered constructs also represent an effective methodology to evaluate the influence of local microenvironments at the metastasis site [42]. Dormancy of tumors is often an important issue that affects early diagnosis and intervention and, hence, the resulting metastasis. Tissue-engineered models, albeit few as of now, are also attempted to evaluate tumor dormancy and reactivation [49]. In vitro models are also useful in these cases to study the behavior of the dormant cells due to the difficulty in obtaining patient samples [50].

Tissue engineering also shows promise in providing therapeutic opportunities for cancer treatment. Patients suffering from post-prostatectomy incontinence or erectile dysfunction due to prostate cancer, or who need reconstructive surgeries, can be treated with tissue engineering therapies [51,52,53]. Bone and other cancers are also treated with the use of tissue-engineered scaffolds [54].

## 3. Cancer Metastasis 3D In Vitro Models

### 3.1. Breast Cancer Metastasis Models

Female breast cancer is reported as the most common cancer and the fourth highest in mortality due to cancer [55]. Cancer originating in the breast can metastasize to the lungs, brain, and bone. Therapies and treatments for bone metastasis of breast cancer are primarily palliative. Metastasis of breast cancer to bone is not curable. The blood-induced mechanical stresses and cancer cell–host (bone) interactions are the major players in bone metastasis of breast cancer. Various material systems are used to develop scaffolds that mimic bone sites. While extensive studies remain underway on the design of primary breast cancer with co-cultures of various cells [56], recent works also report tissue-engineered bone metastasis models. Mimicking the complex dynamic environment of the bone site on arrival of the cancer cells is a useful and valuable approach currently being investigated [57]. Many unique material models have been used to develop tissue-engineered bone structures to evaluate the bone metastasis of breast cancer. These models use a variety of polymeric scaffolds with and without infiltration with bone-forming minerals. A detailed overview of the various materials used to develop the bone niche and cell lines investigated is shown in Table 1. Each material model is tested with in vitro experimentations that validate some hallmarks of breast/prostate cancer colonization to bone.

**Figure 3:**
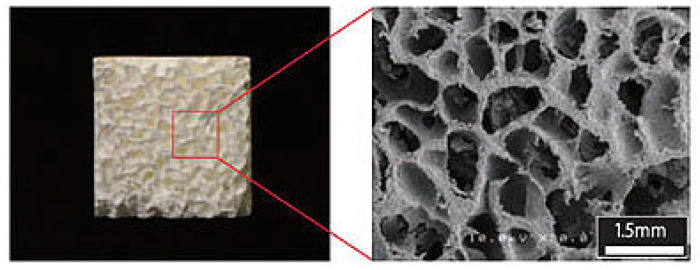
Scanning electron microscope image of hydroxyapatite–collagen composite. Reprinted with permission from [88]. Copyright © 2024 The Japanese Society for Spine Surgery and Related Research.

The bone extracellular matrix (ECM) is considered an attractive site for cancer cell attachment, growth, and survival [84]. Since bone ECM consists of cells, collagen fibers, and hydroxyapatite, extensive studies have been performed using 3D structures fabricated using collagen. Many efforts in the literature involve using collagen fibers to create bone niches. Recent studies use these structures seeded with primary human mesenchymal stem cells and several breast cancer cells: SUM149, SUM159, MDA-MB-231, BT474, MCF7, T47D, and ZR75 [58,59]. Dense collagen hydrogels are fabricated to study the interactions between triple-negative breast cancer cells and bone cells. In particular, the effect of osteolytic breast cancer cells on osteoblast differentiation is studied [60]. Due to the osteogenic characteristic of the mineral hydroxyapatite (HAP), often, collagen composites with HAP are investigated as bone surrogates. Collagen fibers decorated with HAP nanocrystals were made using porcine type 1 atelocollagen [85]. Studies performed with animal models have shown high osteoconductivity and biosorbibility of the HAP-decorated collagen fibers [86,87], as well as the use of these scaffolds to treat osteochondral defects via delivery of paclitaxel to breast cancer bone metastasis in a rat model [88]. This model is potentially useful as an in vitro system but has not been tested as such. The following figure (Figure 3) shows an SEM image of HAP-decorated collagen fibers.

Mammary cells can calcify within the breast tissue, and researchers have utilized the development of an in vitro model of mammary mineralization using murine mammary adenocarcinoma 4T1 cells. These studies make use of collagen-glycosaminoglycan (GAG) scaffolds to mimic the bone environment using murine mammary adenocarcinoma 4T1 cells [61].

Another use of collagen is in a gel form seeded with osteo-differentiated human bone marrow-derived mesenchymal stem cells. This model has successfully used microfluidics to evaluate the extravasation process of the triple-negative and highly metastatic MDA-MB-231 human breast cancer cells [62]. Mineralized collagen fibers were also used for developing a bone-on-a-chip with a co-culture of metastatic breast cancer cells and osteoblasts [63]. A 3D model of the bone niche was fabricated from a collagen matrix (GELFOAM), using endothelial, bone marrow stromal cells, and fetal osteoblasts. This model attempted to evaluate the genes responsible for breast cancer dormancy [59].

Polycapralactone (PCL) is a synthetic biodegradable polyester with a low melting point (~60 °C). PCL is easily degraded by hydrolysis under physiological conditions and is, hence, a commonly used polymer for biomaterial applications. HAP is a commonly used osteogenic ingredient in the scaffold composite. For bone regeneration, PCL infiltrated with various minerals has been used to enhance and/or create osteoinductive, piezoelectric, and strength properties of the polymer. Polycaprolactone (PCL) scaffolds with dispersed HAP have been fabricated using 3D printing to create bone-like models. This in vitro model is used to demonstrate the migration of MDA-MB-231, MCF-7, and MDA-MB-453 breast cancer cells toward the bone [65,66]. The 3D-printed scaffolds made of PCL infiltrated with a piezoceramic barium titanate (BaTiO3) were used to fabricate bone, specifically for load-bearing applications [64]. This bone analog was attempted for use in in vitro models to evaluate MDA MB231 breast cancer cell migration and invasion [41]. Further, microporous PCL scaffolds with polyelectrolyte layers attached to the inner pores were also suggested for use in drug delivery applications [89]. Besides in vitro models, tissue-engineered xenograft models of breast cancer bone metastasis using PCL fibers have recently been attempted [90].

The cancer pre-metastatic niche is composed of ECM proteins (e.g., fibronectin and collagen IV) that play an important role in colonization of cancer cells at the bone niche. In a recent study, microporous ECM protein-coated PCL scaffolds were used to recruit cancer cells in vivo. These models attempted to create a premetastatic niche and were specifically used to evaluate proteins that aide in cancer cell bone metastasis [67]. Researchers have also used random and aligned PCL fibers to mimic the random and organized orientation of collagen fibers in the ECM. Chemo-resistant MDA-MB-231 and T47D breast cancer cells were used to evaluate the efficacy of such an in vitro model, specifically for understanding dormancy in metastasis [68].

**Figure 4:**
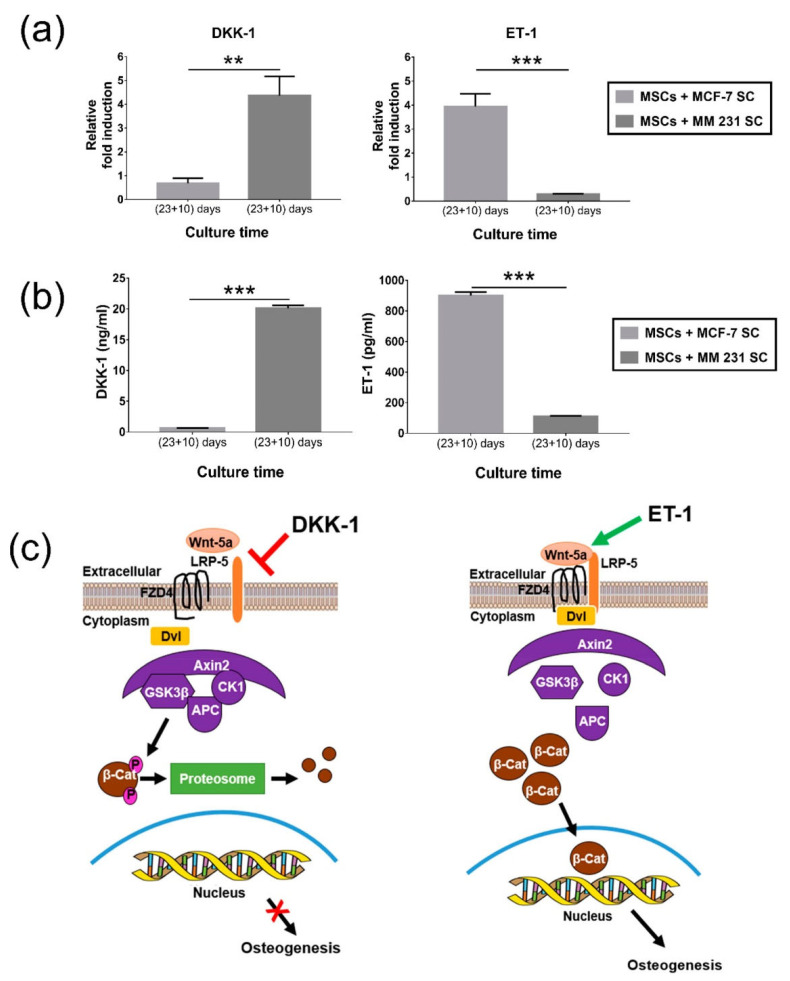
(**a**) Gene expression for breast cancer-related factors, DKK-1 and ET-1, in a bone metastasis in vitro model for MCF-7 and MDA MB231 breast cancer. (**b**) Release of DKK-1 and ET-1 into culture medium. ** *p* < 0.01 and *** *p* < 0.001 indicate significant difference. (**c**) Schematic showing the inactivation and stimulation effects of DKK-1 and ET-1, respectively, on osteogenesis. Reprinted with permission from [74]. Copyright © 2024 American Chemical Society.

PCL infiltrated with nanohydroxyapatite has also been fabricated through a biomimetic process that utilized nano-clay modification with amino acids [91]. Nano-clay–HAP–PCL scaffolds prepared using freeze extraction were used to design in vitro models of breast cancer bone metastasis using a sequential culture of human mesenchymal stem cells and MCF7 and MDA MB231 breast cancer cells [69]. Using this model, the same authors were able to derive mechanics and spectroscopy-based markers of metastasis [71,72], as well as elucidate that the WnT pathway regulates osteogenesis for breast cancer bone metastasis [74] (Figure 4).

The PCL–HAP–nano-clay model was also used with patient-derived breast cancer estrogen-receptor-positive (ER+) and triple-negative (TN) breast cancer tissues to study osteolytic and osteoblastic implications of breast cancer on bone [70] as well as evaluate drug efficacies [73]. Soft gel-like material systems incorporated with nanoHAP were used for 3D-printed structures with vasculature and seeding with multiple cell types to investigate breast cancer metastasis to bone [92]. The choice of gelatin in these models is owing to the fact that gelatin is partially denatured collagen, and the biochemical properties of gelatin are similar to the organic component of bone.

Polyethylene glycol (PEG) is an important hydrophilic polymer commonly used for biomedical applications, due to its excellent biocompatibility, non-immunogenity, and protein repulsion. PEG polymer composites with hydroxyapatite have been attempted as bone biomaterials. Polyethylene glycol hydrogel with nanocrystalline hydroxyapatite is used to make composite scaffolds to mimic the bone native environment. This in vitro model was used to study the interaction between breast cancer cells and osteoblasts [75].

The Kaplan group has pioneered the use of silk proteins for bone tissue engineering [93]. Fibrous proteins derived from natural fibers derived from silkworms and spiders have exceptional mechanical properties. Silk scaffolds seeded with bone marrow stromal cells (BMSC) were implanted in mouse models of human breast cancer metastasis [76]. The silk scaffolds represent a suitable bone niche for metastasis of human breast cancer [76,77,78]. These models have been used to study both breast and prostate cancer bone metastasis.

**Figure 5:**
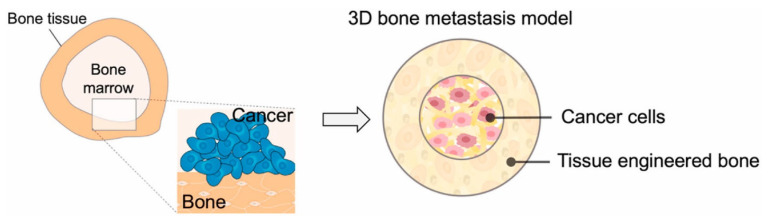
Schematic showing the spatially patterned 3D bone metastasis model. Reprinted with permission from [79]. 0142-9612/© 2024 Elsevier Ltd.

**Figure 6:**
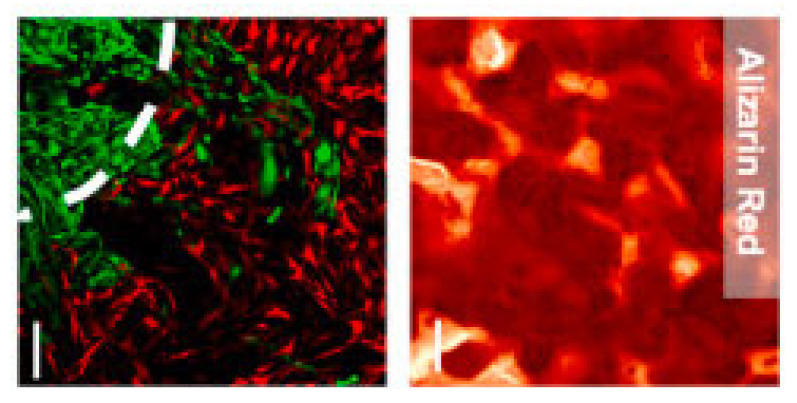
Confocal imaging of breast cancer cell migration into engineered tissues following seven days in co-culture and also alizarin staining to show mineralization. The dashed line denotes the interface with engineered tissues. Scale bar: 200 μm. Reprinted with permission from [79]. 0142-9612/© 2024 Elsevier Ltd.

Other attempts at mimicking the bone site utilize 3D printing to generate a layered structure of scaffold that has an outer ring composed of tissue-engineered bone and a center composed of macro-porous scaffolds that host cancer cells. In a recent study, an innovative design was proposed, wherein a layered structure with an outer layer of tissue-engineered bone and a cancer cell core was fabricated to mimic in vivo metastasis development [79] (Figure 5 and Figure 6).

Polyurethanes are important engineering polymers that have found applications in biomedical engineering due to their biocompatibility, biostability, and degradability. Polyurethane foam scaffolds are investigated as bone surrogates and used as bone metastasis models [80]. The advantage of the PU foam is the apparent highly porous architecture that mimics the trabecular bone. Breast cancer cells, MCF-7-derived tumor-initiating cells (MCFS), were used to evaluate the metastasis condition in these studies.

Similarly, the highly biocompatible and degradable nature of poly (lactide-co-glycolide) (PLA-PGA) polymeric materials makes them useful as bone scaffolds. Scaffolds made using PLA-PGA reinforced with nanoHAP particles were used for the evaluation of adhesion and proliferation of MDA-MB231 breast cancer cells to bone [81,82].

**Figure 7:**
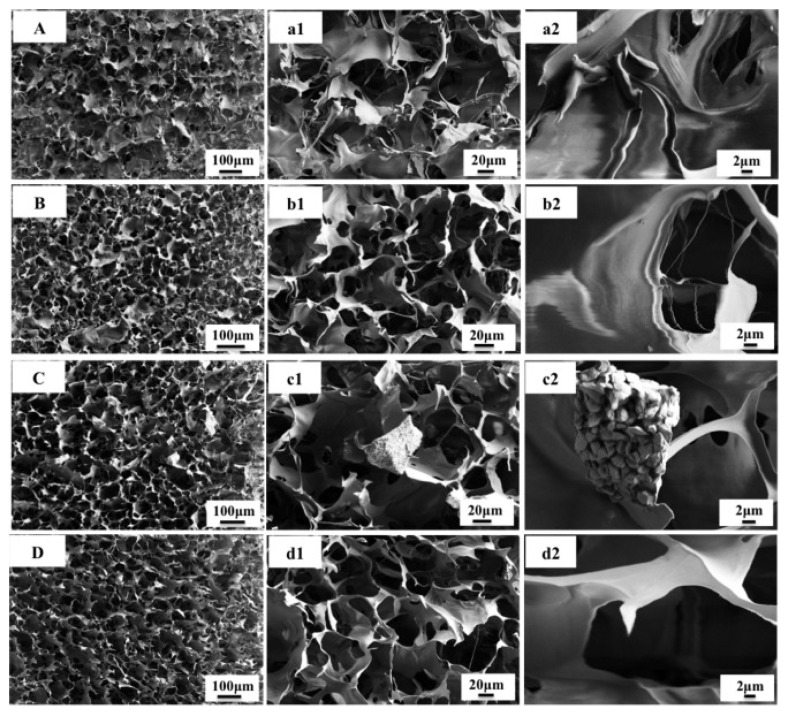
SEM images of bone mimetic scaffolds: (**A**) (**a1**,**a2**) chitosan scaffold control, (**B**) (**b1**,**b2**) nHA chitosan scaffold, (**C**) (**c1**,**c2**) mHA chitosan scaffold, and (**D**) (**d1**,**d2**) amorphous HA chitosan scaffold. Reprinted with permission from [83]. 1742-7061/2014 Acta Materialia Inc. Published by Elsevier Ltd.

**Figure 8:**
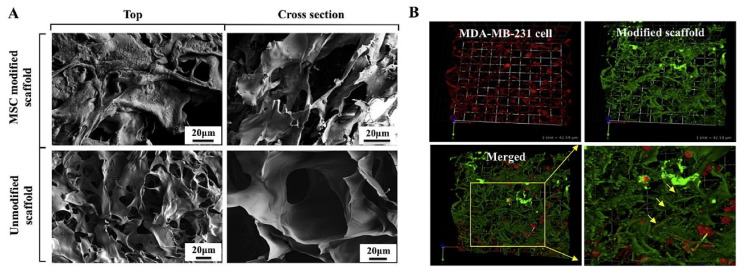
(**A**) SEM images of MSC-modified 10% nHA/chitosan scaffold and unmodified scaffold, compared to unmodified nHA/chitosan scaffold. (**B**) Confocal microscopy images showing the distribution of MDA-MB 231 (red color) cells in the modified scaffold after 24 h. Reprinted with permission from [83]. 1742-7061/2014 Acta Materialia Inc. Published by Elsevier Ltd.

Chitosan is a chitin-derived biopolymer extensively investigated for tissue regeneration and drug delivery applications. The high biocompatibility, degradation properties, and additionally, broad-spectrum antimicrobial qualities, of chitosan drive its applications in tissue engineering. In a recent study, nanoHAP infiltrated inside chitosan gel was used to generate porous bone mimetic scaffolds (Figure 7) [83]. These scaffolds can retain the behavior of less metastatic MCF-7 and highly metastatic MDA-MB231 breast cancer cells (Figure 8) [83].

Further, mimicking breast cancer-induced bone metastasis was also evaluated in vivo using human cancer cells or tissues transplanted into immunocompromised hosts to form xenografts that replicate the bone metastasis [94].

### 3.2. Prostate Cancer Metastasis Models

Prostate cancer cells also metastasize to bone, exhibiting osteomimicry, and are the subject of many investigations [40]. The interactions between the prostate cancer cells and the bone microenvironment are crucial for metastasis progression. Understanding the underlying mechanisms of metastasis of prostate cancer to bone was investigated [95]. Key bone modeling and remodeling process regulatory factors, such as -kappa B (RANK)/RANKL/OPG, the WnT pathways, growth factors, such as TGFb, and specifically, bone morphogenic proteins, are known to be intrinsically involved in the prostate cancer bone metastasis [74,95]. Macro-fluidic models to evaluate the process of metastasis were also attempted [96]. Many attempts have been made to create bone-mimetic environments, such as the bone-mimetic niche for prostate cancer metastasis. For reasons similar to those described for breast cancer metastasis to bone, polycaprolactone scaffolds are fabricated and used for bone scaffolds. Polymers such as PCL and gelatin are also extensively used in bone mimicry for developing bone metastasis models of prostate cancer. Table 2 summarizes the various polymeric, composite, and biological materials that are used in the development of tissue-engineered scaffolds to mimic the bone site of prostate cancer metastasis.

One of the early studies reported the development of a three-dimensional type I collagen gel cell culture system with co-culture of human MG-63 osteoblast-like cells with highly metastatic human PC3 prostate cancer cells. This model was used to study the pathophysiology of prostate cancer at the bone [97].

Recent studies using collagen 3D scaffolds include collagen composites with glycosaminoglycan and nanoHAP [98]. This model was effectively used to study chemosensitivity, cell migration, and proliferation, as well as evaluating the efficacy of delivery of nanoparticle-based gene therapeutics. Other studies reported the use of collagen–nanohydroxyapatite scaffolds containing 5-fold nanohydroxyapatite to collagen by weight, followed by seeding with prostate cancer cells, PC3 and DU145 [102]. New therapeutic studies that evaluate the efficacy of anisamide-targeted amphiphilic cyclodextrin nanoparticles for therapeutic gene silencing are enabled using this model.

Researchers have fabricated scaffolds made using collagen fibers infiltrated with nanoHAP that are further grafted with the glycoprotein SPARC (secreted protein, acidic, and rich in cysteine), known to play a role in bone mineralization. The experiments conducted on the model indicated that the addition of SPARC enabled the survival and growth of the PCa cell line (LNCaP) on the bone-mimetic scaffold.

In the same study where a layered structure with an outer layer of tissue-engineered bone and a breast cancer cell core was fabricated to mimic in vivo metastasis development [79], the authors also used the layered structure for evaluation of prostate cancer metastasis development using PC3 and LNCaP prostate cancer cells. In this study, the authors demonstrated the integration of the layered 3D in vitro model with single-cell RNA sequencing to study fundamental signaling drivers of metastasis [79].

Polycaprolactone is commonly used in bone scaffold development, as described earlier, for developing breast cancer bone metastasis models. A cell-sheet-based technique that consists of wrapping medical-grade polycaprolactone–tricalcium phosphate (mPCL-TCP) scaffolds within hOB sheets was used [103]. An innovative scaffold design consisting of assembly of porcine bone marrow stromal cell (BMSC) cell sheets with medical-grade polycaprolactone–calcium phosphate (mPCL–CaP) scaffolds was utilized to develop bone grafts [105]. Interactions between the prostate cancer cells PC3 or LNCaP with hOBs were investigated. The interactions of PC3 and LNCaP prostate cancer cells with human osteoblasts were studied on these scaffold assemblies [103], followed by animal model experiments [104]. Studies conducted using this model indicate that the prostate cancer cell–bone matrix interactions resulted in elevated levels of metastasis markers, such as elevated MMPs, PSA, and steroidogenic enzymes.

**Figure 9:**
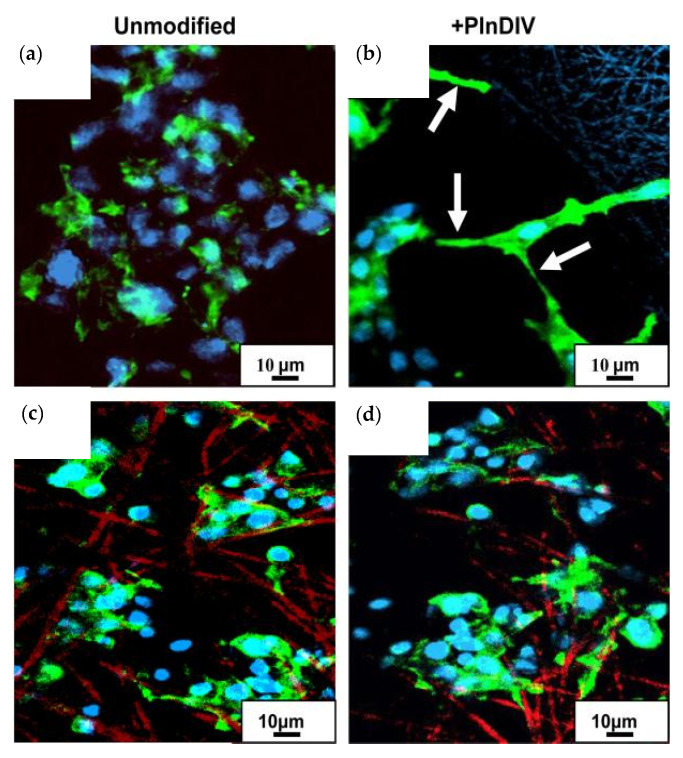
Image showing the reorganization of the cytoskeleton by C4-2B cells on membranes modified by PlnDIV. PCL–gelatin composite membrane with HFIP. (**a**) Unmodified PCL-gelatin composite membrane (**b**) PCL–gelatin composite membrane with HFIP modified with PlnDIV. (**c**) PCL–gelatin composite membrane with TFE. (**d**) PCL–gelatin composite membrane with TFE modified with PlnDIV. HFIP: 1,1,1,3,3,3-hexafluoro-2-propanol; TCE: 2,2,2-trifluoroethanol. Scale bar 10 μm. Reproduced with permission from [107]. 0142-9612/$ e see front matter 2010 Elsevier Ltd.

In a recent work, electrospun PCL fibers and PCL/gelatin composite scaffolds were modified with perlecan domain IV (PlnDIV) peptide and used to develop a pharmacokinetic model to evaluate the proliferation, survival, and migration of C4-2B cancer cells [107] (Figure 9). These studies indicated that PlnDIV peptide plays an important role in the 3D model by helping in the proliferation, survival, and migration of C4-2B cancer cells.

**Figure 10:**
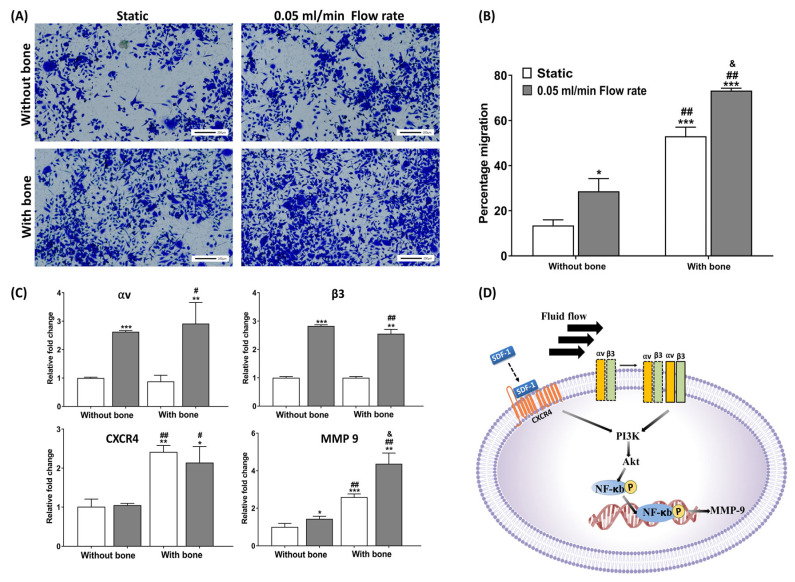
Evaluating the migration of PC3 cancer cells using transwell inserts showing the influence of bone under static and dynamic conditions. (**A**) Cell migration using crystal violet dye under static and dynamic conditions. (**B**) Plot showing the percentage of cell migration in static and dynamic conditions. (**C**) Plot showing the gene expression of genes related to cellular migration. (**D**) Schematic showing the mechanism of the role of CXCR4 and α_v_ and β_3_ integrins in the increase in MMP-9 levels. Here * *p* < 0.05, ** *p* < 0.01, and *** *p* < 0.001 indicating a significant difference between static condition without bone. Similarly, # *p* < 0.05 and ## *p* < 0.01indicating a significant difference between dynamic sample without bone and other conditions. Likewise & *p* < 0.05 indicating a significant difference between the static and dynamic samples with bone. Reproduced with permission from [47]. CC license permission.

As described earlier for breast cancer models, PCL infiltrated with biomimetic nanohydroxyapatite using nano-clay modification with amino acids [91] was also used to develop a prostate cancer bone-mimetic model using a sequential culture of human mesenchymal stem cells, with the highly metastatic PC3 and low metastatic PCa prostate cancer cells [108,109]. This model was also used in combination with perfusion flow and horizontal flow bioreactors, indicating the role of flow-derived shear stresses in the process of metastasis [46,47,48] (Figure 10).

Tubular PCL scaffolds coated with calcium phosphate were fabricated by melt electro-writing PCL and seeded with human osteoprogenitor cells to form bone-mimetic scaffolds [110]. These scaffolds were used to create patient-derived xenograft (PDX) models of lymph node metastasis (LuCaP35) and bone metastasis (BM18) tissues from patients with primary prostate cancer and represent a viable route to derive osteomimicry environments with patient-derived tissues. In a recent study, primary human osteoprogenitor cells were cultured on melt electro-written PCL scaffolds from medical-grade PCL [106]. On these scaffolds, the co-culture of prostate cancer cell lines (LNCaP, C4-2B, and PC3) enables the evaluation of molecular features on these cancer types, as observed in vivo.

The Kaplan group developed an in vitro model using silk proteins derived from Bombyx mori and seeded with prostate cancer cells, PC3 [78]. Experiments conducted with this model indicated that BMP-2 stimulates cancer cell migration. The 3D cultivation of epithelial prostate cancer cells (LNCaP) has also been attempted using silk protein fibroin from Bombyx mori and recombinant spider silk protein spidroin (SSP1) with gelatin, collagen, and chitosan [111], indicating potential advantages.

In a recent work, 3D PLGA and nanohydroxyapatite were fabricated using electro-spraying, compacting, and foaming techniques. These scaffolds were used to evaluate drug toxicity and PC3 cells’ proliferation in bone-like environments [112]. Researchers have also impregnated curcumin into poly(lactic-co-glycolic) acid (PLGA) scaffolds [113]. These studies indicated that curcumin-impregnated PLGA shows increased efficacy against PCa and PC3 bone metastasis using xenograft models.

Co-cultures of human PCa, LNCaP, and human osteoblast cells were seeded onto polyethylene glycol hydrogel scaffolds to study the proliferation of LNCaP prostate cancer cells [114]. These studies illustrate a paracrine effect that promotes osteomimicry and provides insight into the prostate cancer–bone crosstalk. To modulate the mechanical properties of the matrix, researchers have attempted to develop a poly (ethylene glycol)–fibrinogen matrix supplemented with excess poly(ethylene glycol)–diacrylates. In this work, the authors used PC3 prostate cancer cells with BJ-5ta fibroblasts, and presented this new model to study drug treatments and cancer progression [115].

### 3.3. Colon Cancer Metastasis Models

Worldwide, colon cancer remains the fourth most common cancer and the third highest cause of death [55]. Colorectal tumor cells metastasize to the liver and lungs through hematogenous processes or lymphatics. Several attempts to use biomaterials have been made toward controlled drug delivery to colorectal cancer [116,117]. Metastasis is the most common cause of death due to colorectal cancer, with the liver being the most common metastasis site. Due to the high number of fatalities due to colorectal cancer metastasis, there is interest in developing 3D models of metastasis. A detailed description of the material systems and 3D models used to create the metastatic niche of colon cancer is shown in Table 3.

A recent study showed promising results in the use of liver decellularized scaffolds seeded with colorectal cancer cells in mice models [118]. Some researchers have attempted to use decellularized colorectal cancer tissue from biopsies [121] and patient-derived decellularized colon tissue to recapitulate colorectal cancer liver metastasis [119,120]. Generally, decellularized matrices represent the current methodologies in in vitro development of metastasized tissue for the development of new therapeutic agents. Another recent study used decellularized porcine livers to generate scaffolds. On these scaffolds, HCT116 colorectal spheroids were created [122]. In another study, decellularized porcine small intestine submucosa + mucosa was used to create 3D scaffolds that were seeded with SW480 and SW480 colon cancer cells and presented as a tool for testing metastasis mechanisms as well as the efficacy of drugs [123].

**Figure 11:**
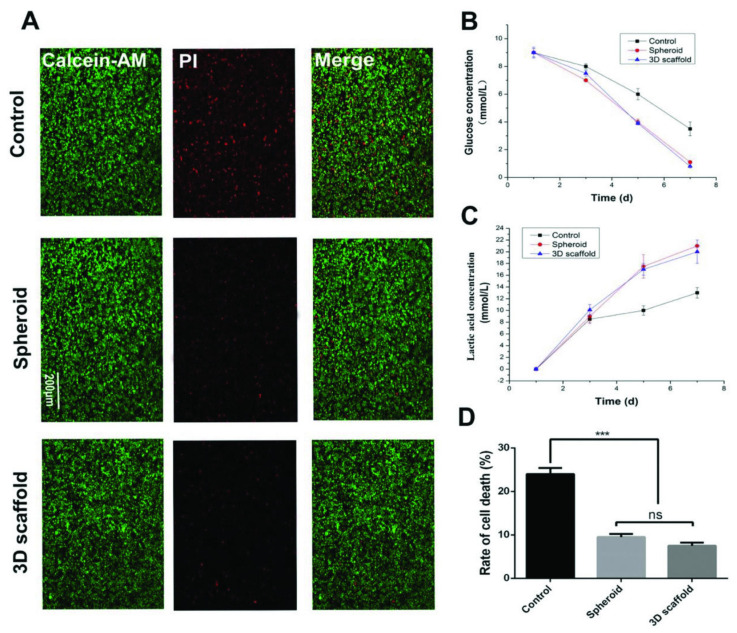
The culture of cancer cells culture on films (control), spheroids, and 3D scaffolds for seven days. (**A**) Fluorescence images of HCT-116 cells cultured on spheroids and scaffolds. Scale bar = 200 µm. (**B**) Plot showing HCT-116 cells’ glucose consumption on spheroids and scaffolds. (**C**) Plot showing lactic acid production by HCT-116 cells cultured on spheroids and scaffolds. (**D**) Rate of cell death in spheroids and scaffolds. *** *p* < 0.005. ns not significant. Reproduced with permission from [124]. © 2024 WILEY-VCH Verlag GmbH & Co. KGaA, Weinheim.

One attempt at creating a synthetic polymeric biomaterial scaffold involved scaffolds prepared using E-jet 3D printing of PLGA seeded with HCT-116 and LoVo human colon cancer cell lines, as well as the p53-null (knockout) human colon cancer cell line (HCT-116 p53^−/−^) [124]. The authors demonstrated the use of this scaffold system for evaluating the key role of p53 in cellular migration responsible for metastasis (Figure 11).

Although the advantages of a true metastatic niche using tissue engineering are known, these attempts are limited to liver metastasis of colon cancer. In vivo models have been studied to evaluate the metastasis potential of colon cancer cells [125,126,127,128]. Many recent studies have focused on use of patient-derived organoids for evaluation of metastatic potential [129]. Tissue engineering and biomaterials are needed to create 3D in vitro models of colorectal cancer metastasis [130]. There is an unmet need to develop liver mimetic tissue-engineered scaffolds to evaluate metastasis therapeutics and colon cancer progression.

### 3.4. Pancreatic Cancer Metastasis Models

Although the incidence of pancreatic cancer is lower, ranking fourteenth in cancer incidence worldwide, mortality is 94% of the incidence [55]. The common sites of metastasis of pancreatic cancer are the liver (76–80% of patients), peritoneum (48%), and the lungs (45%) [131]. The liver metastasis of pancreatic cancer is a multistage and multistep process. The primary pancreatic tumor is highly invasive and, hence, in vitro models are mainly developed at the primary site. Mouse models have traditionally been extensively studied to understand the pathobiology of pancreatic cancer tumors [132].

The in vitro models of pancreatic cancer create the primary site of cancer. The 3D organoid structures are currently being investigated to evaluate the pathology and migration of primary site pancreatic cancer [133,134]. Recently, 3D organoids developed from patient tissue sources or genetically engineered mouse models have been developed as tools for patient-specific therapies of primary site pancreatic cancer and are suitable as preclinical models of pancreatic cancer [135]. Decellularized tissue scaffolds have also been attempted for use as viable models of late-stage pancreatic cancer. In one study, decellularized human pancreas and livers were seeded with PANC-1 and MIA PaCa-2 cell lines and PK-1 cells (liver-derived metastatic pancreatic cancer cell line) [136]. A novel recent study used tissue engineering approaches with a porous polyurethane scaffold modified with fibronectin and seeded with pancreatic cancer cells (PANC-1). In vitro hypoxia was administered in this model to study the impact of radiotherapy treatment [137].

## 4. Comparative Differences between the Various In Vitro Models of Metastasis

Use of decellularized tissues and complex polymeric and composite scaffolds has been attempted for many models, as shown in Table 1, Table 2 and Table 3. The ready availability and manufacturability of the polymeric and composite models make them more likely candidates for clinical applications. The other important variable is the complex array of co-cultures needed in the scaffolds. While many studies point to the in vitro models needing the whole array of cell types at the metastasis site, there is increasing evidence that mimicking some of the characteristics of migration, osteogenesis (in particular for the bone metastasis), and MET characteristics is adequate to build in vitro models that may be used for clinical applications of personalized medicine design with ease of manufacturability and low cost.

## 5. Emerging Areas and Future Perspectives

Due to the significant economic and human impacts of cancer metastasis worldwide, extensive efforts are undertaken for developing tissue-engineered complex constructs and co-cultures simulating the metastasis site, as demonstrated in this review. Two emerging areas can be identified that present a promising future avenue for the use of in vitro models for treatment and understanding of cancer metastasis. These areas, in combination with the development of realistic cellular co-culture models with patient-derived cell lines, present a very optimistic future for the use of in vitro cancer metastasis models. The first area is the use of hydrodynamic considerations in tissue growth. While many of the studies in the development of accurate 3D models that recapitulate the metastasis site are being conducted, recent developments in cell culture under fluid flow conditions have shown significant importance. Various bioreactor designs have been attempted to provide physiologically relevant flow velocity and velocity contours through the culture media during cellular growth and proliferation in tissue engineering [138]. These bioreactors provide the important input of fluid-derived shear stresses that are known to greatly impact cell proliferation and metastasis [46,48]. Bioreactor experiments also indicate that the scaffold interstitial flow-induced shear stress is a critical factor for enabling migration of cancer cells at their extravasation stage [47]. Incorporation of tissue-engineered bioreactor-enabled 3D in vitro models are likely to be the next-generation models for evaluation of metastasis potential, as well as a route toward personalized medicine. Additionally, the use of microfluidics approaches shows promise in potential for developing cancer-on-a-chip models [139,140]. With the advances in tissue engineering technologies, more effective cancer-on-a-chip models that effectively capture the tumor microenvironment nuances and flow characteristics will be developed.

The second area of potential high impact involves using computational tools to evaluate metastasis. Modeling studies, in particular multiscale models of tissue engineering scaffolds, are gaining acceptance due to their ability to reproduce in vivo effects [141,142,143,144,145]. As a result, in silico oncology approaches also show promise [146,147,148,149,150,151]. With developments in exa-scale computing, real-time oncology modeling is a real possibility [151]. The use of tissue engineering scaffolds as therapies for cancer is also a promising area of application, as a source of new engineered tissue [152]. Recently, a new area has evolved that attempts to use tissue-engineered cancer metastasis as a therapy, using it as a cancer vaccine [153]. Further, spatial transcriptomics has arisen as a new option to characterize the vast spatiotemporal heterogeneity of cancers [154]. While this technique benefits diagnosis and predictions, future studies will likely use in vitro scaffold models for spatial transcriptomics investigations.

Thus, while significant advances have been accomplished in some areas of metastasis models, e.g., bone sites for prostate and breast cancer, other 3D models are also gaining importance. Reduced use of animal models with the help of advanced predictable in vitro systems is likely to speed up research development of methods to combat metastasis and provide reconstruction therapies to patients. Many material systems and processes were investigated, as reviewed here, and generally, each presented new insights toward understanding metastasis, the heterogeneity of cancer types, and the spatiotemporal existence of cancer metastasis in patients. It is unlikely that a single material system will prove to be the magic key for metastasis, but several unique material scaffolds will be utilized for patient-specific treatments.

## Figures and Tables

**Table 1:** List of materials and cells for developing the bone niche for breast cancer bone metastasis models.

Material Class	Material Type	Cell Types	References
Collagens	Collagen fibers and hydroxyapatite	HUVEC (human umbilical cord endothelial cells) Breast cancer cells tested: SUM149, SUM159, MDA-MB-231, BT474, MCF7, T47D, ZR75	[58,59]
	Dense collagen hydrogel	MDA-MB-231 breast cancer cells and MC3T3-E1 pre-osteoblasts	[60]
	Collagen-glycosaminoglycan (GAG)	Murine mammary adenocarcinoma 4T1 cells	[61]
	Collagen gel seeded with osteo-differentiated human bone marrow-derived mesenchymal stem cells	MDA-MB-231 human breast cancer cells	[62]
	Heavily mineralized collagen fibers for a bone-on-a-chip	A co-culture of metastatic breast cancer cells and osteoblasts GFP-labeled metastatic breast cancer cell line, MDA-MB-231GFP cells, and metastasis-suppressed breast cancer cell line, MDA-MB-231-BRMS1GFP cells	[63]
	3D Collagen matrix (GELFOAM), seeded with endothelial, bone marrow stromal cells, and fetal osteoblasts	MDA-MB-231, BoM1833	[59]
PCL	3D-printed scaffolds made of Piezo-electro-ceramics, such as BaTiO_3_ with polycaprolactone	In vitro models of MDA MB231 breast cancer cell migration and invasion studies	[41,64]
	3D-printed polycaprolactone (PCL) scaffolds with dispersed HAP	This in vitro model shows migration of MDA-MB-231, MCF-7, and MDA-MB-453 breast cancer cells toward the bone	[65,66]
	PCL scaffolds coated with fibronectin and collagen IV	Human LM2-4 cells derived from MDA-MB-231 cells and mouse 4T1 cells	[67]
	Random and aligned PCL fibers	Chemo-resistant MDA-MB-231 and T47D breast cancer cells	[68]
	PCL with nano-clay–biomimetic hydroxyapatite	MCF 7, MDA 231, patient-derived cell lines	[69,70,71,72,73,74]
PEG	Polyethylene glycol hydrogel and nanocrystalline hydroxyapatite composite scaffolds	MDA-MB-231	[75]
Silk Proteins	Fibrous proteins derived from natural fibers derived from silkworms and spiders	MDA MB 231, MCF 7	[76,77,78]
	Silk protein scaffolds	Human breast cancer cells injected into the mammary fat pads of mice	[49,50,51,77]
	3D-printed spatially layered bone tissues with gelatin to generate a layered structure of scaffold that has an outer ring composed of tissue-engineered bone and a center composed of macroporous scaffolds that host cancer cells	MDA MB 231	[79]
Polyurethane	Polyurethane foam scaffold	MCF7	[80]
PLA-PGA	Poly (lactide-co-glycolide) PLA-PGA scaffolds dispersed with nanoHAP particles	MDA-MB231	[81,82]
Chitosan	NanoHAP inside a chitosan gel	MDA-MB-231, MCF-7, and transfected MDA-MB-231	[83]

**Table 2:** List of materials and cells for developing the bone niche for prostate cancer bone metastasis models.

Materials System	Material Form	Cell Types	References
Collagens	Collagen gel	Co-culture of human MG-63 osteoblast-like cells with highly metastatic human PC3 prostate cancer cells	[97]
	Collagen-glycosaminoglycan and nanohydroxyapatite composites	PC3 and LNCaP	[98]
	Collagen nanofibers with nanohydroxyapatite grafted with SPARC	LNCaP	[99,100,101]
	Collagen-hydroxyapatite scaffolds	PC3 and DU145	[102]
Gelatin	3D printing to generate a layered structure of scaffold that has an outer ring composed of tissue-engineered bone and a center composed of macroporous scaffolds that host cancer cells	PC3	[79]
PCL	Medical-grade polycaprolactone–calcium phosphate (mPCL–CaP) scaffolds	PC3 and LNCaP	[103,104,105]
	Medical-grade PCL: culturing primary human osteoprogenitor cells on melt electrowritten PCL scaffolds	LNCaP, C4-2B, and PC3	[106]
	Electrospun PCL fibers and PCL/gelatin composite scaffolds modified with perlecan domain IV (PlnDIV) peptide	C4-2B cancer cells	[107]
	PCL–nano-clay–nanohydroxyapatite scaffolds	PC3 and PCa	[91,108,109]
	Tubular PCL scaffolds coated with calcium phosphate were fabricated by melt electro-writing PCL	LuCaP35	[110]
Silk Proteins	Scaffolds fabricated from silk proteins derived from Bombyx mori.	PC3	[78]
	Scaffolds fabricated using silk protein fibroin from Bombyx mori and recombinant spider silk protein spidroin (SSP1) with gelatin, collagen, and chitosan, indicating potential advantages	LNCaP	[111]
PLA-PLGA	PLGA and nanohydroxyapatite scaffolds	PC3	[112]
	Curcumin-impregnated poly(lactic-co-glycolic) acid (PLGA) scaffolds		[113]
PEG	Polyethylene glycol hydrogel	PCa and LNCaP	[114]
	Scaffolds fabricated with poly(ethylene glycol)-fibrinogen matrix supplemented with poly(ethylene glycol)-diacrylate	PC3 with BJ-5ta fibroblasts	[115]

**Table 3:** List of materials and cells for developing the colon cancer metastasis models.

Material Class	Material Form	Cell Types	References
Decellularized tissue scaffolds	Liver decellularized scaffolds seeded with colorectal cancer cells in mice models	HT-29, CRC119, SW480, and Caco2	[118]
	Patient-derived decellularized colon tissue	HT-29	[119,120,121]
	Decellularized porcine livers to generate scaffolds	HCT116	[122]
	Decellularized porcine small intestine submucosa + mucosa scaffolds	SW480 and SW480 colon cancer cells	[123]
PLGA	E-jet 3D printing of PLGA	HCT-116 and LoVo human colon cancer cell lines, and p53-null (knockout) human colon cancer cell line (HCT-116 p53^−/−^)	[124]

## Data Availability

No new data were collected in this review article.
